# The proxy problem anatomized: child-parent disagreement in health related quality of life reports of chronically ill adolescents

**DOI:** 10.1186/1477-7525-10-10

**Published:** 2012-01-25

**Authors:** Jane NT Sattoe, AnneLoes van Staa, Henriëtte A Moll

**Affiliations:** 1 Rotterdam University, Expertise Centre Transitions of Care, P.O. Box 25035, 3001 HA Rotterdam, the Netherlands; 2 Erasmus University Rotterdam-Institute of Health Policy & Management, P.O. Box 1738, 3000 DR Rotterdam, the Netherlands; 3 Erasmus MC University Medical Center-Sophia Children's Hospital, Department of Paediatrics, P.O. Box 2060, 3000 CB Rotterdam, the Netherlands

**Keywords:** Adolescent, Chronic Illness, Self Report, Quality of Life, Parent, Proxy Report, KIDSCREEN-10, DCGM-10

## Abstract

**Background:**

Discrepancy between self-reports and parent-proxy reports of adolescent health-related quality of life (HRQoL) has been repeatedly acknowledged in the literature as the proxy problem. However, little is known about the extent and direction of this discrepancy. The purpose of this study is to explore to what extent and in what direction HRQoL self-reports of adolescents with chronic conditions and those of their parents differ.

**Methods:**

A cross-sectional survey was conducted among adolescents suffering from chronic conditions and their parents. Socio-demographic and disease-related characteristics were collected and information about consequences of the chronic condition was assessed. HRQoL was measured with KIDSCREEN-10 and DISABKIDS condition generic measure (DCGM-10). Agreement was analysed through defining a threshold of agreement based on half of the standard deviation of the HRQoL score with the highest variance. Agreement occurred if the difference between adolescent and parent scores was less than or equal to half of the standard deviation. Intra-class correlation coefficients and Bland-Altman plots were also computed. The characteristics associated with direction of disagreement were statistically tested with one-way ANOVA and Chi-square tests.

**Results:**

584 paired HRQoL scores were obtained. Ratings from both adolescents and parents were high, compared to European norm data. Differences between adolescents and parents were statistically significant, yet relatively small. Disagreement existed in both directions: in 24.5% (KIDSCREEN-10) and 16.8% (DCGM-10) of the cases adolescents rated their HRQoL lower than did their parent, while in 32.2% (KIDSCREEN-10) and 31.7% (DCGM-10) of the cases the opposite was true. Adolescent's age, educational level and type of education, parent's educational level, number of hospital admissions and several other disease-related factors influenced direction of disagreement.

**Conclusions:**

In a reasonable proportion of cases the adolescent and parent agreed on the adolescent's HRQoL (43-51% of the cases) and most disagreement tended to be minor. Thus, the proxy problem may be smaller than presented in the literature and its extent may differ per population. As adolescents are expected to become partners in their own health care, it is recommended to focus on adolescents' own perceptions of HRQoL.

## Background

Paediatric care professionals have been debating whether parent proxy reports of their children's Health Related Quality of Life (HRQoL) are reliable enough [1,2]. Since both patient and parent-proxy reports are often used in paediatric and adolescent care, discrepancies between the two may complicate the use of HRQoL information in clinical practice-for instance, when determining if complementary interventions are needed [3].

Discrepancies between child HRQoL reports and parent proxy reports have repeatedly been acknowledged in the literature as 'the proxy problem' [1,2,4,5], but little is known about influencing factors [2,6-9] and the direction of discrepancy [10,11]. A systematic review about child-parent agreement in HRQoL reports that agreement is influenced by the child's age, gender and health status. However, no consistent conclusions about the direction and extent of influence of these factors could be derived [1].

White-Koning et al. [12] evaluated Quality of Life (QoL) reports of children with cerebral palsy and their parents and found that the following factors influenced agreement: disease severity, the family's socioeconomic status, parental characteristics, and the absence of behavioural problems. They also found that the child's gender did not independently seem to affect child-parent agreement, a finding confirmed by various other studies [13-17]. Most studies on child-parent (dis)agreement, however, focus on specific diagnoses and younger children. The question arises to what extent these results hold for chronically ill adolescents and their parents more generally.

Gaining more insight into child-parent disagreement is particularly valuable in the field of adolescent care. An important goal for care for chronically ill adolescents is preparing the transition from paediatric to adult care. Transition requires good self-management competencies and skills [18]. A first step in enhancing these adolescents' self-reliance is to explore how they evaluate their chronic condition. It also seems important to find out how parents think about their children's health, because parental perception can influence the child's use of health care services [4] and parents are expected gradually to relinquish their care giving responsibilities to their child [7,18].

The aim of this study is to explore to what extent and in what direction HRQoL self-reports of adolescents with somatic chronic conditions and those of their parents differ, and to study associated factors.

## Methods

### Population

The data in this study are derived from a study among adolescents with chronic conditions and their parents recruited from a university children's hospital in the Netherlands, focusing on adolescents' preferences and competencies for health care and self-management (reported elsewhere [19,20]). This sub-study focused on the comparison of adolescent and parent ratings of HRQoL.

The target group consisted of all adolescents aged 12-19 years suffering from a somatic chronic condition or physical impairment, who were treated in the departments of Paediatrics or Paediatric Surgery at Erasmus MC-Sophia Children's Hospital, Rotterdam, the Netherlands. More specifically: they must have consulted the outpatient clinic at least three times or must have been hospitalized at least once in the three years prior to July 1^st ^2006. Exclusion criteria were the following: transfer to adult care already effected or documented diagnosis of intellectual impairment.

Eligible adolescents and their parents received written information about the study and were invited to complete a web-based questionnaire accessible for three months (October-December 2006) with a unique code on a secured Internet site.

Response cards were included to encourage adolescents to state, if this should be the case, that they did not qualify for the study, or to explain why they did not wish to participate. All potential participants received a reminder after three weeks. There was no financial remuneration, although participants were entered in a lottery for two iPods and a cell phone.

Approval was obtained from the Erasmus MC Institutional Review Board. Participants were assured of confidentiality and data were processed anonymously. The researchers had no access to participants' medical records.

### Measures

The parent version of the questionnaire was constructed as a mirror version of the adolescent version (i.e. parents were asked to rate presumed adolescents' perceptions).

#### Main outcome variables

Respondents completed the generic short forms of the European KIDSCREEN questionnaire (KIDSCREEN-10) [21] and the European DISABKIDS condition generic measure (DCGM-10) [22,23]. We chose the short versions to reduce the time respondents needed to fill in the questionnaires. Proxy versions are available for both questionnaires. The KIDSCREEN-10 questionnaire is validated to assess HRQoL in both healthy and chronically ill adolescents and children and provides a singular index of global HRQoL [21,24]. Its 10 items are all scored on a 5-point scale ranging from 'never/not at all' to 'always'. The item scores are combined into a final score on a scale from 0 to 100 [21].

The DISABKIDS condition generic measure was designed to document the HRQoL of children and adolescents and to describe the impact of a disease on their wellbeing [22,23,25]. The chronic generic short version assesses HRQoL aspects related to being ill in general. It consists of 12 Likert-scaled items assigned to mental, social and physical domains of HRQoL. The items are scored on a 5-point scale ranging from 'never' to 'always'. Ten items produce a score on a scale from 0 to 100 [25]. Two items are related to the use of medication and are not included in the final score.

The availability of both an adolescent and a parent version and the good psychometric properties of the questionnaires were important reasons for choosing the KIDSCREEN-10 and the DCGM-10 questionnaires. The developers report a good internal consistency: Cronbach's alpha is .82 for the child version of the KIDSCREEN-10 and .82 for the parent version. The reported concordance between the parent and child version is also good, with a Pearson coefficient r = .73 [21]. For the DCGM-10 the reported Cronbach's alpha is .84 for the child version and .86 for the parent version, with a Pearson coefficient r = .82 [25].

#### Socio-demographic characteristics, disease-related characteristics and consequences of the condition

Adolescents' age and gender were retrieved from the hospital database. Educational level (higher, indicating preparation for higher education, versus lower) and type of education (regular education versus special education for the physically disabled) of adolescents and parents were informed after in the questionnaire. Because ethnicity is not recorded in the hospital database, the family names were manually classified by two independent researchers into Dutch versus non-Dutch, using the Dutch Databank of Surnames. This method has shown good reliability in other studies [26,27].

Health care-related characteristics such as the number of outpatient consultations, hospital admissions and the different outpatient departments visited between July 1^st ^2003 and June 30^st ^2006 were retrieved from the hospital database. Age at diagnosis (0-5 years, or after the age of 5) and absenteeism from school or work due to illness in the past year were assessed in the questionnaire by asking how often a day at school or work had been missed (1-item question on a 5-point Likert scale; range: 1 = never, 2 = sometimes, 3 = regularly, 4 = often, 5 = very often). Adolescents and parents also provided information on any therapeutic regimen (i.e. medication, diet or exercises) prescribed to the adolescent. Adolescents' limitations in mobility and independence were measured with the Activities of Daily Living Tool (AVO-99 [28]). The original 10-item scale was dichotomized: if any physical limitation was present, this was recorded as 1.

The experienced burden of the visibility of the condition was measured through a combination of two questions in each questionnaire. These questions were "Can other people see that you are/your child is disabled?" (range: 1 = never, 2 = sometimes, 3 = regularly, 4 = often, 5 = very often/always) and "How annoying is this for you/your child?" (range: 1 = not annoying at all, 2 = not annoying, 3 = a little annoying, 4 = annoying, 5 = very annoying). The sum score of these questions in both versions of the questionnaire was computed by adding up the two ratings. This led to a variable with a theoretical range between 2 and 10 [19].

#### Statistical analysis

SPSS 17.0 (SPSS Inc, Chicago, IL) was used for all the statistical analyses. Means, standard deviations and proportions were used for descriptive analyses. McNemar tests were used to test for differences between adolescent and parent reports of dichotomous disease-related factors. Paired Samples t-tests were performed to test whether the reported means of the continuous disease-related factors differ significantly between adolescents and parents. Paired Samples t-tests were also performed to test differences in means of HRQoL between adolescents and parents. To study the direction of agreement between adolescent self-reports and parent-proxy reports, agreement was established according to the definition of clinically meaningful difference in quality of life [29]. Agreement was assumed to occur when the absolute difference between the scores of adolescents and their parents was less than or equal to 0.5 SD of the score with the largest variability (this group is referred to as AGREE). Disagreement was also based on computing difference scores and was defined to occur if adolescents rated their HRQoL lower (this group is referred to as ADOL LOW) or higher (this group is referred to as ADOL HIGH) than did their parents-indicated by a difference in rating that is higher than the threshold for agreement. The extent of disagreement was classified into four levels: from 0.5 to 1 SD (minor), from 1 to 1.5 SD (intermediate), from 1.5 to 2 SD (major), and higher than 2 SD (substantial). Alternatively, Bland-Altman plots [30] were computed to study the extent of disagreement and intraclass correlation coefficients (ICCs) were computed to identify any disagreement between adolescents and their parents.

One-way ANOVA and Chi-square tests served to study the demographic, health care- and disease-related factors associated with the direction of agreement. In addition, Tukey post-hoc tests and Chi-square post-hoc tests with Bonferonni correction were applied. Variables were considered significant predictors at p < .05 and all the statistical tests were two-tailed.

## Results

### Studied population

We obtained 584 paired adolescent-parent responses (53.7% of the net adolescent response and 68.1% of the net parent response). Analyses revealed that non-responders were more frequently males and had non-Dutch surnames; they were older and less frequent visitors to the hospital compared to responders (p < .05). In the study sample, the five largest diagnostic categories (ICD-9 classification) were: congenital anomalies and conditions originating in the perinatal period (31%); neoplasm (13%); endocrine, nutritional, metabolic diseases, and immunity disorders (12%); diseases of the nervous system and sense organs (11%); and diseases of the musculoskeletal system and connective tissue (33%). Table 1 presents the socio-demographic characteristics of the adolescents and their parents, the disease-related characteristics of the adolescents measured through both the adolescent and parent questionnaire, and the health care-related characteristics, retrieved from the hospital database (including the five largest ICD-9 diagnostic groups). The differences in adolescent and parent perceptions turned out to be significant for two of the four disease-related factors (Table 1).

**Table 1:** Description of the study sample according to respondent, No. (%), n = 584 (unless indicated)

	Adolescents	Parents	p*
***Socio-demographic characteristics***			
**Gender**			
Female	322 (55.1)	303 (54.7)	
Male	262 (44.9)	251 (45.3)	-
**Age**			
12-15	371 (63.5)		
16-19	213 (36.5)	-	-
*Mean (SD)*	14.9 (1.9)		
**Educational level**			
Lower	326 (56.2)	338 (59.7)	-
Higher	254 (43.8)	228 (40.3)	
Education type**			
Regular	526 (90.7)	-	-
Special	54 (9.3)		
**Ethnicity**			
Dutch surname	526 (90.7)	-	-
Non-Dutch surname	54 (9.3)		
***Disease-related characteristics***			
**Age at diagnosis**			
0-5 yrs	428 (73.3)	-	-
≥ 6 yrs	156 (26.7)		
**Number of visits of outpatient department**			
*Range*	1-111	-	-
*Mean (SD)*	16.9 (15.4)		
**Number of hospital admissions**			
*Range*	0-138	-	-
*Mean (SD)*	4.9 (9.8)		
**Number of different outpatient departments**			
*Range*	1-15	-	-
*Mean (SD)*	3.1 (2.2)		
***Consequences of chronic condition ***			
**Presence therapeutic regimen**			
Yes	378 (64.7)	386 (66.1)	ns
**Presence physical limitations**			
Yes	165 (28.3)	133 (22.9)	< .01
**School/work absenteeism**			
*Range*	1-5	1-5	ns
*Mean (SD)*	1.9 (.90)	1.9 (.85)	
**Experienced burden**			
*Range*	2-10	2-10	< .01
*mean (SD)*	4.6 (2.1)	5.2 (2.1)	

* McNemar test or Paired Samples t-test to test if the reported variables differ significantly between adolescent reports and parent reports.

** n = 580

Since our analysis concerned a selection of all adolescents and parents that participated in the study, we performed additional independent samples Mann-Whitney U tests and t-tests to compare the study sample with the excluded sample. The mean HRQoL did not significantly differ between adolescents for whom parent-proxy reports were available and the other adolescents. The same was true for mean age, gender and educational level. The excluded sample contained a higher proportion of adolescents with non-Dutch surnames and of adolescents who were six years or older when their condition was diagnosed. More details of this analysis are presented in an additional file [see Additional file 1]. The tests were repeated between parents for whom adolescent self-reports were available and other parents. The only significant difference here was that the former group contained a higher proportion of mothers.

### Health Related Quality of Life

Table 2 provides ranges, means, standard deviations, medians, and interquartile ranges of scores on the KIDSCREEN-10 and DCGM-10 scales.

**Table 2:** Main outcome variables, n = 584

	KIDSCREEN-10	DCGM-10
Range	17.5-100	16.7-100
No. of items	10	10
Mean (SD) Adolescents	78.2 (15.6)*	80.2 (16.3)**
Median Adolescents	80.0	83.3
Interquartile Range Adolescents	22.5	19.4
Mean (SD) Parents	76.9 (16.0)*	76.5 (17.6)**
Median Parents	80.0	77.8
Interquartile Range Parents	22.5	27.8

*p < .05 in Related Samples Wilcoxon Signed Ranks test to test if the means differ significantly between adolescent and parent reports.

**p < .01 in Related Samples Wilcoxon Signed Ranks test to test if the means differ significantly between adolescent and parent reports.

On average, adolescents scored their HRQoL higher than did their parents. The mean scores of adolescents were respectively 78.3 (SD = 15.6) and 80.2 (SD = 16.3) for KIDSCREEN-10 and DCGM-10. The mean scores of parents were respectively 76.8 (SD = 16.1) and 76.4 (SD = 17.7) for KIDSCREEN-10 and DCGM-10.

The adolescents' median scores were 80.0 and 83.3 for KIDSCREEN-10 and DCGM-10, respectively. These are similar to the Dutch norm data. The medians in the norm data were 77.5 for KIDSCREEN-10 and between 82.5 and 85.0 for DCGM-10. Compared to the European norm data, our mean KIDSCREEN-10 score was higher, but the standard deviation in our sample was similar. The norm score was 71.9 (SD = 15.0).

The Cronbach's alpha was .64 for the child version of the KIDSCREEN-10 self-report and .71 for the parent version. The Cronbach's alphas of the DCGM-10 questionnaire were satisfactory values (child version: .82 and parent version: .87). The degree of correlation between the KIDSCREEN-10 HRQoL score and the DCGM-10 HRQoL score was considerable. The Pearson correlation coefficient was .57 for adolescents and .68 for parents (both p < .01).

**Figure 1:**
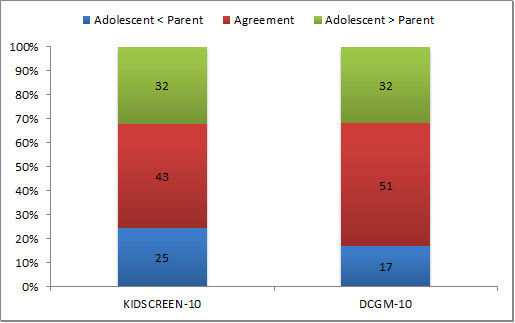
**Distribution of agreement between adolescent and parent reports (percentage of complete pairs)**. Agreement = adolescent-parent score ≤ .5 greatest SD of scores, i.e. the threshold for respectively KIDSCREEN-10 and DCGM-10: 8 points, 9 points.

The Paired Samples t-tests showed statistically significant differences between the adolescents' and parents' scores (p < .05; Table 2). For KIDSCREEN-10 the mean difference was 1.3 (SD = 17.1); for DCGM-10 the mean difference was 3.7 (SD = 15.1). The threshold for agreement was around 8 points for the KIDSCREEN-10 HRQoL scores and around 9 points for the DCGM-10 HRQoL scores. Figure 1 represents the distribution of agreement between adolescent and parent reports. For KIDSCREEN-10, 43% of the adolescent-parent pairs agreed with each other. For DCGM-10 this was 51%. Disagreement occurred in either direction. The ICC (using an absolute agreement definition) for the KIDSCREEN-10 measure was .42; for the DCGM-10 measure it was .59. Both were significant (p < .01), indicating that there is agreement about adolescent HRQoL between adolescents and their parents.

### Extent of disagreement

**Figure 2:**
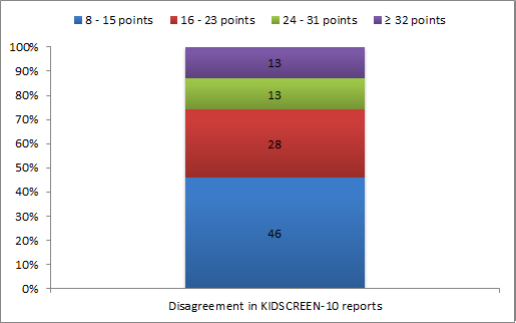
**Distribution of disagreement in KIDSCREEN-10 reports (percentage of complete pairs)**. Disagreement = adolescent-parent score > respectively 0.5, 1.0, 1.5, and 2.0 times the SD of the HRQoL score with the highest variability.

**Figure 3:**
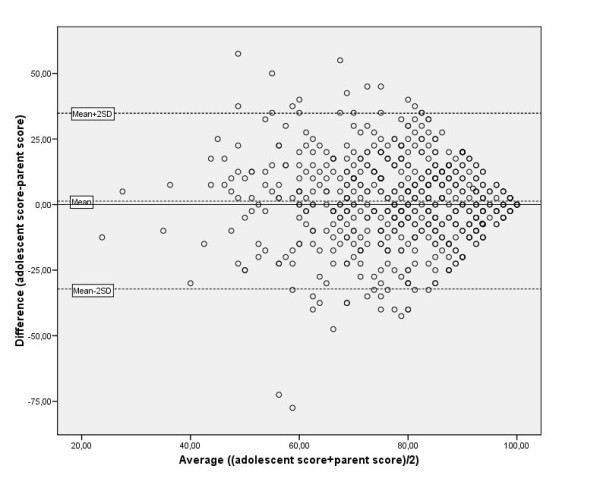
**Adolescent-parent agreement in KIDSCREEN-10 reports**. Bland-Altman analysis: mean difference (SD) = 1.3 (17.1).

Taking the threshold of agreement for KIDSCREEN-10 as 8 points, four levels to explore the extent of disagreement were defined: minor: 8-15 points (0.5-1 SD); intermediate: 16-23 points (1-1.5 SD); major: 24-31 points (1.5-2 SD); and substantial: 32 or more points (2 SD or higher). Almost half of the disagreement in KIDSCREEN-10 reports was minor; 28% was intermediate; 13% was major; and 13% was substantial (Figure 2). The mean difference between adolescent and parent reports was 1.3 (SD = 17.1); most adolescent-parent pairs fell within the agreement limits in the Bland-Altman plot (Figure 3).

**Figure 4:**
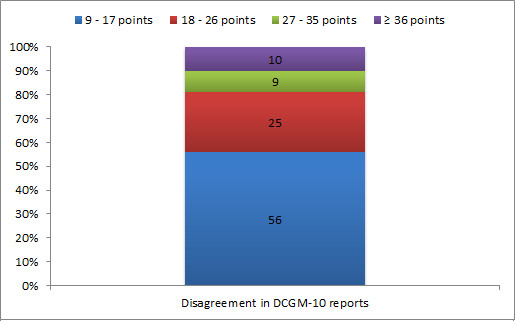
**Distribution of disagreement in DCGM-10 reports (percentage of complete pairs)**. Disagreement = adolescent-parent score > respectively 0.5, 1.0, 1.5, and 2.0 times the SD of the HRQoL score with the highest variability.

**Figure 5:**
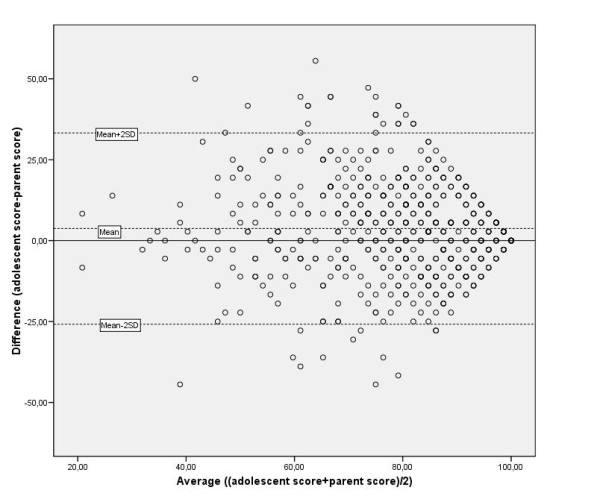
**Adolescent-parent agreement in DCGM-10 reports**. Bland-Altman analysis: mean difference (SD) = 3.7 (15.1).

The threshold of agreement for DCGM-10 was 9 points. The levels of disagreement were respectively: minor: 9-17 points (0.5-1 SD); intermediate: 18-26 points (1-1.5 SD); major: 27-35 points (1.5-2 SD); and substantial: 36 or more points (2 SD or higher). Fifty-six percent of the disagreement in DCGM-10 reports was minor; 25% was intermediate; 9% was major; and 10% was substantial (Figure 4). The mean difference between adolescent and parent reports was 3.7 (SD = 15.1); most adolescent-parent pairs fell within the agreement limits in the Bland-Altman plot (Figure 5).

### Direction of disagreement

Three groups of (dis)agreement were defined: ADOL LOW, AGREE and ADOL HIGH, and differences between these groups were tested with one-way ANOVA tests and Chi-square tests.

#### KIDSCREEN-10

With respect to the rating of global HRQoL, the three groups significantly differed on several demographic characteristics of the adolescent: age, educational level and type of education; and on adolescents' disease-related characteristics as perceived by their parents: physical limitations, school/work absenteeism and experienced disease burden. The results are presented in Table 3.

**Table 3:** KIDSCREEN-10 results, mean (SD) or No. (%), n = 584 (unless indicated)

	ADOL LOW	AGREE	ADOL HIGH	df_M_	df_R_	F or H	*p*
Socio-demographic characteristics*							

**Gender (A)**							
Female	91 (63.2)	132 (52.4)	99 (52.7)	2	-	4.73	ns
**Gender (P)**							
Female	85 (61.6)	124 (52.3)	94 (52.5)	2	-	3.37	ns
**Age (A)**	15.3 (1.9)^a^	14.8 (1.9)	14.8 (1.9)	2	581	3.63	< .05
**Educational level (A)**							
Lower	76 (52.8)	128 (51.4)	122 (65.2)^b^	2	-	9.25	< .05
**Educational level (P)**							
Lower	86 (61.9)	146 (60.1)	106 (57.6)	2	-	.552	ns
**Education type (A)**							
Regular	134 (93.1)^c^	233 (93.6)	159 (85.0)^b^	2	-	9.83	< .01
**Ethnicity (A)**							
Dutch surname	134 (93.1)	225 (89.3)	177 (94.1)	2	-	4.33	ns

Disease-related characteristics*							

**Age at diagnosis (A)**							
Before age of six	111 (77.1)	173 (68.7)	144 (76.6)	2	-	5.64	ns
**No. of outpatient visits**	15.8 (12.5)	16.4 (14.6)	18.4 (18.1)	2	581	1.31	ns
**No. hospital admissions**	4.3 (6.1)	4.9 (10.7)	5.4 (10.8)	2	581	.494	ns
**No. different outpatient departments**	2.9 (1.9)	3.0 (2.1)	3.5 (2.6)	2	581	3.36	ns

Consequences of chronic condition*							

**Therapeutic regimen (A)**							
Yes	87 (60.4)	163 (64.7)	128 (68.1)	2	-	1.92	ns
**Therapeutic regimen (P)**							
Yes	88 (61.1)	166 (65.9)	132 (70.2)	2	-	3.15	ns
**Physical limitations (A)**							
Yes	42 (29.2)	60 (23.8)	63 (33.5)	2	-	5.32	ns
**Physical limitations (P)**							
Yes	19 (13.4)^c^	52 (20.6)	62 (33.2)^b^	2	-	19.02	< .01
**School/work absenteeism (A)**	1.9 (.87)	1.8 (.87)	2.0 (.97)	2	581	2.03	ns
**School/work absenteeism (P)**	1.8 (.78)^d^	1.8 (.80)	2.1 (.94)^a^	2	577	7.01	< .01
**Experienced burden (A)**	4.7 (2.2)	4.5 (2.1)	4.6 (2.1)	2	580	.733	ns
**Experienced burden (P)**	5.2 (2.3)	5.0 (2.1)	5.5 (1.9)^a^	2	581	3.07	< .05

^a^A Tukey post-hoc test revealed that this group differed significantly from the agreement group AGREE on a p < .05 level.

^b^A Chi-square post-hoc test with Bonferonni correction revealed that this group differed significantly from the agreement group AGREE on a p < .017 level.

^c^A Chi-square post-hoc test with Bonferonni correction revealed that this group differed significantly from the disagreement group ADOL HIGH on a p < .017 level.

^d^A Tukey post-hoc test revealed that this group differed significantly from the disagreement group ADOL HIGH on a p < .01 level.

*(A) stands for information assessed in the adolescent questionnaire, while (P) stands for information coming from the parent questionnaire.

Post-hoc tests revealed that adolescents in the ADOL LOW group (15.3, SD = 1.9, p < .05) were significantly older than those in the AGREE group (14.8, SD = 1.9, p < .05) and that a lower educational level was more common in the ADOL HIGH group (65.2%) than in the AGREE group (51.4%; p < .017). Furthermore, special education was more common in the ADOL HIGH group (15.0%) than in the AGREE group (6.4%) and in the ADOL LOW group (6.9%; p < .017). The presence of a physical limitation, as perceived by the parent, was more likely in the ADOL HIGH group (33.2%) versus both the AGREE group (20.6%; p < .017) and the ADOL LOW group (13.4%; p < .017). School/work absenteeism as perceived by parents was significantly higher in de ADOL HIGH group (2.1, SD = .94, p < .01) than in the AGREE group (1.8, SD = .80, p < .01) and in the ADOL LOW group (1.8, SD = .78, p < .01). Finally, the experienced disease burden (as perceived by parents) in the ADOL HIGH group (5.5, SD = 1.9) was higher than that in the AGREE group (5.0, SD = 2.1, p < .05).

#### DCGM-10

With respect to the impact of the chronic condition on the adolescent's HRQoL, the (dis)agreement groups differed on educational level of both the adolescent and the parent, the number of hospital admissions and on disease-related characteristics as perceived by parents: presence of physical limitations and experienced burden of the condition. Results are presented in Table 4.

**Table 4:** DCGM-10 results, mean (SD) or No. (%), n = 584 (unless indicated)

	ADOL LOW	AGREE	ADOL HIGH	df_M_	df_R_	F or H	*p*
Socio-demographic characteristics*							

**Gender (A)**							
Female	55 (56.1)	163 (54.2)	104 (56.2)	2	-	.243	ns
**Gender (P)**							
Female	51 (54.8)	155 (54.2)	97 (55.4)	2	-	.068	ns
**Age (A)**	14.8 (1.9)	14.9 (1.9)	15.0 (1.9)	2	581	.192	ns
**Educational level (A)**							
Lower	65 (67.0)^b^	153 (51.3)	108 (58.4)	2	-	7.82	< .05
**Educational level (P)**							
Lower	66 (72.5)^bc^	168 (57.5)	104 (56.8)	2	-	7.42	< .05
**Education type (A)**							
Regular	89 (91.8)	272 (91.3)	165 (89.2)	2	-	.744	ns
**Ethnicity (A)**							
Dutch surname	88 (83.7)	274 (91.0)	165 (94.1)	2	-	2.00	ns

Disease-related characteristics*							

**Age at diagnosis (A)**							
Before age of six	82 (83.7)	212 (70.4)	134 (72.4)	2	-	3.14	ns
**No. of outpatient visits**	16.6 (12.8)	16.2 (14.4)	18.3 (18.0)	2	581	1.09	ns
**No. hospital admissions**	3.9 (5.8)	4.2 (5.9)	6.6 (15.0)^a^	2	581	4.04	< .05
**No. different outpatient departments**	3.0 (2.0)	3.1 (2.3)	3.4 (2.3)	2	581	1.38	ns

Consequences of chronic condition*							

**Therapeutic regimen (A)**							
Yes	62 (63.3)	188 (62.5)	57 (30.8)	2	-	2.38	ns
**Therapeutic regimen (P)**							
Yes	65 (66.3)	190 (63.1)	131 (70.8)	2	-	3.03	ns
**Physical limitations (A)**							
Yes	28 (28.6)	88 (29.2)	49 (26.5)	2	-	.433	ns
**Physical limitations (P)**							
Yes	14 (14.4)^c^	63 (21.1)	56 (30.3)	2	-	10.20	< .01
**School/work absenteeism (A)**	1.8 (.79)	1.9 (.97)	1.9 (.85)	2	581	.801	ns
**School/work absenteeism (P)**	1.7 (.72)	1.9 (.89)	2.0 (.84)	2	577	2.83	ns
**Experienced burden (A)**	4.8 (2.3)	4.6 (2.1)	4.6 (2.1)	2	580	.640	ns
**Experienced burden (P)**	4.9 (2.1)^d^	4.9 (2.1)	5.8 (2.0)^a^	2	581	12.27	< .01

^a^A Tukey post-hoc test revealed that this group differed significantly from the agreement group AGREE on a p < .05 level.

^b^A Chi-square post-hoc test with Bonferonni correction revealed that this group differed significantly from the agreement group AGREE on a p < .017 level.

^c^A Chi-square post-hoc test with Bonferonni correction revealed that this group differed significantly from the disagreement group ADOL HIGH on a p < .017 level.

^d^A Tukey post-hoc test revealed that this group differed significantly from the disagreement group ADOL HIGH on a p < .01 level.

*(A) stands for information assessed in the adolescent questionnaire, while (P) stands for information coming from the parent questionnaire.

Post-hoc tests revealed that a lower educational level of the adolescent was more common in the ADOL LOW group (67.0%) than in the AGREE group (51.3%; p < .017). A lower parent educational level was also more common in the ADOL LOW group (72.5%) versus both the AGREE group (57.5%) and the ADOL HIGH group (56.8%; p < .017).

The number of hospital admissions was higher in the ADOL HIGH group (6.6, SD = 15.0, p < .05) versus the AGREE group (4.2, SD = 5.9, p < .05). A physical limitation, as perceived by the parent, was more likely in the ADOL HIGH group (30.3%) versus the ADOL LOW group (14.4%; p < .017). Finally, the disease burden (as perceived by the parent) was significantly higher in the ADOL HIGH group (5.8, SD = 2.0) versus both the AGREE group (4.9, SD = 2.1, p < .01) and the ADOL LOW group (4.9, SD = 2.1, p < .01).

## Discussion

This study investigated the extent and direction of disagreement between HRQoL reports of adolescents with a variety of somatic chronic conditions and their parents in a sample of 584 pairs. About half of the pairs agreed on adolescents' HRQoL. For the other pairs, statistically significant disagreement in either direction was found. Yet, the differences were relatively small (respectively 74% (KIDSCREEN-10) and 81% (DCGM-10) of the adolescent-parent disagreement was minor or intermediate). The ICCs and Bland-Altman plots also indicated reasonable agreement between adolescents and parents.

Our results would suggest that the 'proxy problem' of child-parent disagreement in HRQoL evaluations is perhaps not as meaningful as is often assumed in the literature. For example, White-Koning et al. [12] found a higher rate of disagreement (64%) than we did (respectively 57% and 48% for KIDSCREEN-10 and DCGM-10). They also defined agreement in terms of a clinically meaningful difference in quality of life. HRQoL was measured with the KIDSCREEN-52 questionnaire and their population size was comparable to ours [12]. However, White-Koning et al. studied 8-12-year-old children with cerebral palsy, whereas we studied 12-19-year-old adolescents with a variety of chronic conditions. So it seems plausible that the size of the proxy problem may depend on disease category and age group. Shaw et al. [11], for instance, found a rate of disagreement in a population of adolescents with juvenile idiopathic arthritis (JIA) that is consistent with our findings, while Ylimainen et al. [31] found poor agreement between parent and child reports of the child's HRQoL in young persons with limb reduction deficiency. Next to this, the small thresholds of agreement in our study, 8 and 9 points respectively for KIDSCREEN-10 and DCGM-10, are additional arguments to question the size of the proxy problem, because they indicate little variance in HRQoL. Most of the disagreement we found was minor.

Yet, a considerable proportion of adolescents and parents disagreed with each other on HRQoL. In these cases, the adolescent usually reported a higher HRQoL. This is consistent with previous studies in children with chronic conditions [4,10,16,17,32,33]. Conversely, a minority of parents rated their child's HRQoL higher than did the adolescents themselves, which has not often been reported in the literature on chronically ill adolescents [4].

In our study, adolescents who disagreed with their parents on both global HRQoL and HRQoL related to the impact of a chronic condition were more likely to have a lower educational level than those who agreed with their parents. An explanation could perhaps be found in social status differences, which are seen to be related to the differential ways that parents and children rate health [32]. The same explanation could hold for our finding that parents with a lower educational level are more likely to overestimate their child's HRQoL instead of agreeing with their child or underestimating the HRQoL.

Regarding age, Cremeens et al. [33] and Majnemer et al. [34] found that agreement increased with increasing age of the adolescent. In our study, however, adolescents agreeing with their parents were more often younger than the ones who rated their global HRQoL lower than did their parents. This conflicting finding may perhaps be explained by the fact that the aforementioned studies did not correct for direction of disagreement. Previous findings on direction of disagreement mostly focused on the ADOL HIGH group [4,32,34]. The discrepancy between findings is plausible since the effect of age was evident only when comparing the ADOL LOW group with the AGREE group. Parents were more likely to overestimate HRQoL of older adolescents. The differences in age of adolescents were minute, indicating that even a few months in this crucial period of adolescence make a difference. Perhaps parents saw older adolescents as more capable when it comes to living with a chronic condition. As another explanation, parents may be less well informed about their child's wellbeing at adolescent age, implicating that health care providers would do well to focus on the opinions of the adolescents themselves.

Adolescents who rated their HRQoL higher than their parents did, scored less well on the health-care related and disease-related factors (interpreted by the parents) than did all other adolescents. Parents seem to attach greater value to these factors. Our finding is consistent with literature findings indicating that disease-severity factors are associated with child-parent disagreement [10,35]. This is also seen in the cases of adolescents who rated their HRQoL lower than did their parents. These adolescents' parents perceived fewer physical limitations, lower school absenteeism, and lower experienced burden than the parents that underestimated their child's HRQoL. Perhaps the differences in HRQoL perception could in part be explained by the discrepancy in adolescents' perception and parents' perception of the impact of the condition on quality of life. As an additional argument, the correlation between the proxy versions of KIDSCREEN-10 and DCGM-10 is higher than that between the child versions, indicating that parents perceive a stronger relation between general HRQoL and the HRQoL related to impact of the condition than adolescents do. Gates et al. [36] also found that parents focus more on functional aspects than adolescents do. While adolescents tend to focus on their abilities, the parent's perspective is more likely one of disability [37]. Therefore, adolescent self-reports and parent-proxy reports of HRQoL are not interchangeable. Furthermore, given that parents of chronically ill children themselves report seriously lower HRQoL compared to controls [38], and parental wellbeing is known to influence (proxy) measurement of HRQoL [12], assessing parents' own HRQoL is perhaps more meaningful than asking them for a proxy report of their child.

Finally, the adolescents' mean HRQoL score was higher than the European norm score for KIDSCREEN-10 [21]-despite the fact that all adolescents were chronically ill. The descriptive statistics indicate a ceiling effect, which may be ascribed to the so-called 'disability-paradox' explaining "why many people with serious and persistent disabilities report that they experience a good or excellent quality of life when to most external observers these individuals seen to live an undesirable daily existence" [39]. This paradox implies that HRQoL for persons with disabilities is broader than just health, encompassing the person's social context and environment too. Perhaps our population benefited from a positive and supportive social environment. Next to this, adaptation (a phenomenon referred to as response shift) [40] cannot be ruled out. The majority of our adolescent population has lived with their condition for almost all of their conscious life.

### Strengths and limitations

Our study included a large sample of adolescents with a wide range of chronic conditions. The sample was heterogeneous in terms of congenital and acquired conditions, and in age. It originates from the largest university hospital in the Netherlands, which comprises all major pediatric subspecialties. Yet the wide range of chronic conditions made it impossible to explore the impact of nature of the disease and that of disease severity. This diversity in chronic conditions may also be responsible for the wide standard deviations in both adolescent and parent reports.

However, since chronically ill adolescents all face the same adaptive challenges [41], studying chronic conditions in general is not considered a flaw. Disease severity, however, is a broad concept that can be operationalised in different ways. In this study, we included only health-care and disease-related variables into the models but no psychological measures. Certain psychological factors, such as child-parent conflict, could have had an effect on the extent and direction of disagreement [42]. Also, the short forms of the used HRQoL questionnaires do not allow for analyses at the level of the different HRQoL domains. There are indications that child-parent (dis)agreement is dissimilar in these domains [43]-for example, one study established more disagreement for the mental (psychological) domain compared to the physical and social domains [12]. Analyses of (dis)agreement at the level of specific domains could have provided further insight in the spread and nature of (dis)agreement in our study population. For further research, we recommend using the longer versions to be able to test for differences between the HRQoL domains.. Furthermore, the non-response rate was fairly high (63%). More information on the sample and the non-response is reported elsewhere [19]. Candidates received an impersonal letter and were required to access the questionnaire on the Internet. The returned response postcards made clear that many candidates did not feel 'chronically ill'. Apart from this, lay views on 'being ill' and the importance of 'being normal' may have played a role here. Non-responders consulted the hospital less frequently than did responders, which may imply that they represent a healthier population, although it may also indicate no-show. The non-response analysis revealed that notably older adolescents, boys and adolescents with non-Dutch surnames were underrepresented. This might have affected the outcomes. It is impossible, however, to tell in what way. Adolescents excluded from analysis because there was no proxy questionnaire available, more often had a non-Dutch surname. An explanation for this finding might be that non-Dutch parents were facing more language and cultural barriers than Dutch parents when asked for participation in (HRQoL) research. This has been reported before in Turkish and Moroccan ethnic minority patients in the Netherlands [44]. However, little is known about child-parent disagreement in ethnic minorities. Therefore it is impossible to tell if, and how, this finding affected the outcomes of the study. The same is true for our finding that excluded adolescents more often reported having received a diagnosis after the age of six. There were no significant differences between the total sample of parents and our sub-sample of parents, with the exception of gender: the sub-sample included more mothers. This is the case in most of the comparable studies [1]. The effect of parent gender on HRQoL assessment, however, is unknown [1].

## Conclusions

In this sample of chronically ill adolescents and their parents any disagreement was predominantly minor, which raises questions about the size of the proxy problem. However, in around 20% of all cases adolescents and parents disagreed to a greater extent. Parents tended to underestimate their child's HRQoL, but still a reasonable number overestimated it. Parents' and adolescents' educational level and adolescent's age should be taken into account when interpreting HRQoL-reports. Parents seem to weigh the impact of the condition more heavily than their child does, indicating that self-reports and parent-proxy reports are not interchangeable. However, since adolescents are expected become partners in their own health care and HRQoL measures provide relevant clinical information about psychosocial functioning, it is recommended to focus on the adolescent's own perceptions of HRQoL.

## List of abbreviations

*ADOL HIGH*: group of cases in which parents underestimate their child's HRQoL compared with the adolescent's rating; ADOL *LO*W: group of cases in which parents overestimate their child's HRQoL compared with the adolescent's rating; *AGREE*: group of cases in which adolescents and their parents agree about the adolescent's HRQoL; *HRQoL*: Health Related Quality of Life; *ICC*: intra class correlation; *SD*: standard deviation; *QoL*: Quality of Life.

## Competing interests

The authors declare that they have no competing interests.

## Authors' contributions

JNTS carried out the literature study, performed the statistical analysis and drafted the manuscript. AvS conceived the study, participated in its design and coordination and helped to draft the manuscript. HAM contributed to the analysis and interpretation of the data, and critically revised the manuscript for important intellectual content. The On Your Own Feet Research Group participated in the design and execution of the study. All authors read and approved the final manuscript.

## Supplementary Material

Additional file 1 **Comparison of the study sample with the sample excluded from analysis**. A table presenting the results of an additional analysis recommended by the reviewer. Click here for file
