# Supplementary material for: The proxy problem anatomized: child-parent disagreement in health related quality of life reports of chronically ill adolescents
Source: Health Qual Life Outcomes. 2012 Jan 25;10:10. doi: 10.1186/1477-7525-10-10 (PMC3299605; doi:10.1186/1477-7525-10-10)
Supplement: Additional file 1 — Comparison of the study sample with the sample excluded from analysis. A table presenting the results of an additional analysis recommended by the reviewer. [file 1477-7525-10-10-S1.PDF]

## *Comparison of the study sample with the sample excluded from analysis, No. (%) or Mean (SD)*

| | **Adolescents included in study sample N=584** | **Adolescents excluded from analysis N=455** | ***p**** |
|---|---|---|---|
| ***Socio-demographic characteristics*** | | | |
| **Gender** | | | |
| Female | 322 (55.1) | 263 (57.8) | ns |
| Male | 262 (44.9) | 192 (42.2) | |
| **Age** | | | |
| 12 – 15 | 371 (63.5) | 268 (58.9) | ns |
| 16 – 19 | 213 (36.5) | 187 (41.1) | |
| *Mean (SD)* | 14.9 ( 1.9) | 15.1 ( 2.0) | |
| **Educational level**[a,b] | | | |
| Lower | 326 (56.2) | 202 (55.2) | ns |
| Higher | 254 (43.8) | 164 (44.8) | |
| **Ethnicity** | | | |
| Dutch surname | 526 (90.7) | 351 (77.1) | <.001 |
| Non-Dutch surname | 54 ( 9.3) | 104 (22.9) | |
| ***Disease-related characteristics*** | | | |
| **Age at diagnosis**[c] | | | |
| 0-5 yrs | 428 (73.3) | 282 (62.8) | <.001 |
| ≥6 yrs | 156 (26.7) | 167 (37.2) | |
| **Number of visits of outpatient department** | | | |
| *Range* | 1-111 | 1-146 | ns |
| *Mean (SD)* | 16.9 (15.4) | 17.6 (17.9) | |
| **Number of hospital admissions** | | | |
| *Range* | 0-138 | 0-139 | ns |
| *Mean (SD)* | 4.9 ( 9.8) | 4.4 (10.1) | |
| **Number of different outpatient departments** | | | |
| *Range* | 1-15 | 1-15 | ns |
| *Mean (SD)* | 3.1 ( 2.2) | 3.1 (2.3) | |
| ***Consequences of chronic condition*** | | | |
| **Presence therapeutic regimen**[d] | | | |
| Yes | 378 (64.7) | 259 (60.0) | ns |
| **Presence physical limitations**[e] | | | |
| Yes | 165 (28.3) | 129 (28.9) | ns |
| **School/work absenteeism** | | | |
| *Range* | 1-5 | 1-5 | ns |
| *Mean (SD)* | 1.9 ( .90) | 2.0 ( .93) | |
| **Experienced burden** | | | |
| *Range* | 2-10 | 2-10 | ns |
| *mean (SD)* | 4.6 ( 2.1) | 4.8 ( 2.3) | |

* Independent samples Mann-Whitney U test to test if distributions or Paired-Sample t-test to test if the means differ significantly between study sample and excluded sample.

[a] N=580 for study sample.

[b]N=366 for excluded sample.

[c]N=449 for excluded sample.

[d]N=432 for excluded sample.

[e]N=447 for excluded sample.
